# A Rare Case of Atherosclerosis of the Angular Artery Manifested as a Nodular Lesion on the Skin and Review of the Literature

**DOI:** 10.1155/crid/6001887

**Published:** 2025-02-07

**Authors:** Wender Rodrigues Nazário, Marcondes Pedro Souza Novais, Anaíra Ribeiro Guedes Fonseca Costa, Débora de Oliveira Santos, Douglas Magallhães de Paula, Sérgio Vitorino Cardoso, Paulo Rogério de Faria

**Affiliations:** ^1^Department of Oral and Maxillofacial Pathology, Federal University of Uberlândia, Uberlândia, Brazil; ^2^Department of Integrated Clinics, University Center of Patos de Minas, Patos de Minas, Brazil

**Keywords:** angular artery, atheroma, atherosclerosis, face, facial artery branches, histopathological analysis

## Abstract

Atherosclerosis is an age-related disease affecting the tunica intima of large and medium-sized arteries. Its occurrence in extracranial head and neck arteries is rare, with only five cases reported. This study is aimed at reporting the sixth case of atherosclerosis of the angular artery, a branch of the facial artery, in an elderly male patient with a history of hypertension. Morphological characterization using special stains and a literature review are included. Clinically, a painless, nodular lesion measuring 1.2 cm was observed at the right labial commissure. The lesion was excised, and atherosclerosis of the angular artery was diagnosed. After 6 months, there was no recurrence or other arterial issues. Despite its rarity in the oral and maxillofacial region, dental clinicians must be aware of this condition and its clinical manifestations, especially in elderly patients with chronic systemic diseases, to establish an accurate diagnosis.

## 1. Introduction

The facial artery, which originates from the external carotid artery, exhibits significant anatomical variations in its course, branches, and terminations [1]. It usually emerges above the lingual artery and traverses the submandibular region, where anomalies in its course through the submandibular gland have been reported [1]. Its trajectory continues medially and anteriorly, supplying the face, with notable variations in its branching pattern, including the presence of triple premasseteric branches [2, 3]. The artery's termination also varies, as it may end near the angular artery or form alternative connections with other facial vessels [4]. Rarely, its origin deviates to an intraparotid location, highlighting the variability in its anatomical presentation [5]. Even rare, this artery and its branches may be affected by cardiovascular diseases. Hence, identifying variations in branching patterns is clinically relevant, aiding surgeons in delicate procedures involving this region and treating such vascular lesions.

Atherosclerotic cardiovascular disease is a slow-growing, age-associated arterial lesion characterized by tunica intima thickening due to cholesterol and cholesterol ester, collagen and elastic fibers, smooth muscle cell, and inflammatory cell (T lymphocytes and monocyte-derived macrophages) accumulation causing a gradual narrowing, hardening, and flow-limiting stenosis of the affected artery [6–8]. Necrosis and necrosis-associated dystrophic calcification are other alterations that may also develop inside the atherosclerotic plaques over time, leading to more stiffness of the affected vessel and, thus, cardiovascular complications [8]. Cigarette smoking, unhealthy diet, physical inactivity, diabetes mellitus, dyslipidemia, and hypertension are the major causal factors for atherosclerosis development and progression [9, 10].

Atherosclerotic lesions of extracranial head and neck arteries are extremely rare compared with other large elastic and large- to medium-sized muscular arteries of the body [11, 12]. However, when present in the head and neck, such lesions frequently involve the external carotid arteries and their branches, especially temporal and facial arteries [13, 14]. The atherosclerosis-affected facial artery usually manifests as a painless, firm nodule or tumor-like mass in the skin of systemic chronic disease–harboring patients [13, 15]. Some lesions may also develop true aneurysms because of the vessel wall weakness [13, 16]. Here, we describe the first case report of atherosclerosis of the angular artery, a facial artery branch, in an elderly patient that manifested as a painless nodule in the skin. We also characterized morphologically the lesion with special stains and reviewed the English literature.

## 2. Case Presentation

**Figure 1:**
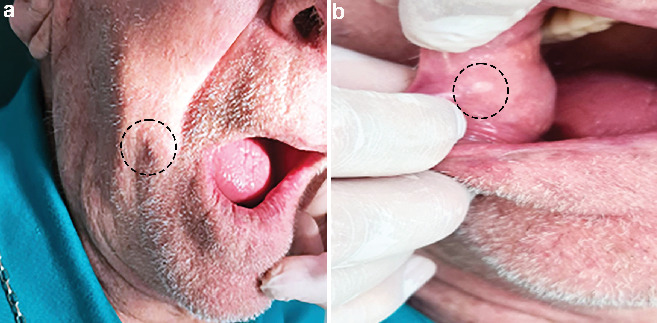
(a) A nodular lesion is present on the skin of the right labial commissure (dashed circle). (b) The intraoral aspects of the lesion cause whitish discoloration of the buccal mucosa (dashed circle).

An 86-year-old white male sought a private dental clinic complaining of a painless nodule in the face that had lasted for 5 years. During anamnesis, he reported that the nodule rapidly increased in size until its current dimension was achieved. The patient was in good health condition (American Society of Anesthesiologist (ASA) II classification), and his medical history only included controlled hypertension. He denied diabetes and history of trauma or surgery to the region. The patient's familial medical history was unremarkable. The clinical examination revealed a rubbery, firm, mobile, 1.5-cm nodule in the face near the right labial commissure (Figure 1(a)). Intraoral examination showed that the lesion caused a well-circumscribed, whitish macule in the buccal mucosa (Figure 1(b)). Two months later, he underwent a blood examination, but no abnormalities were found. Based on these observations, the clinical diagnosis of an epidermoid cyst was established, and the lesion was planned to be excised immediately.

**Figure 2:**
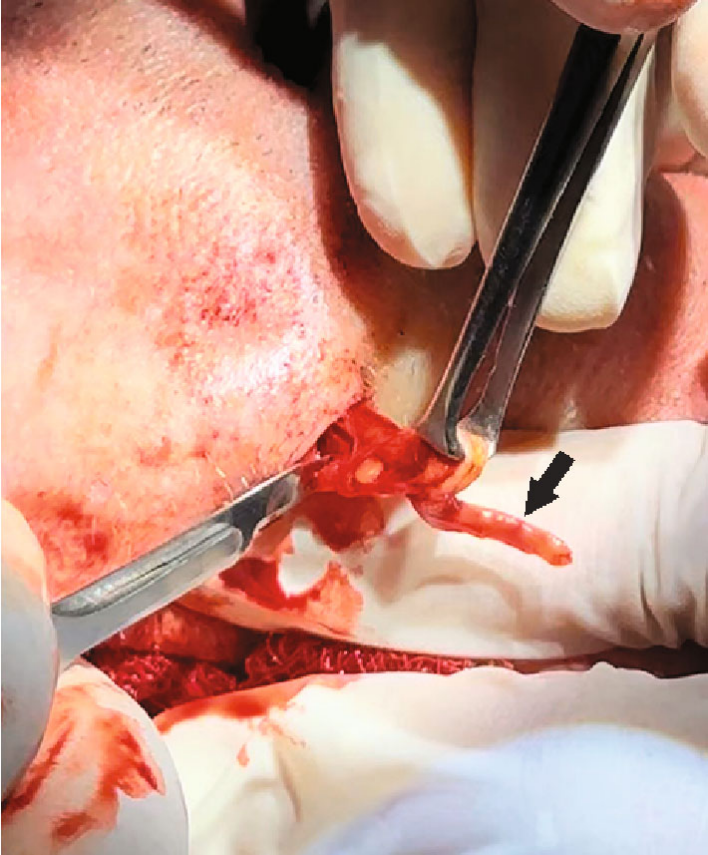
Surgical procedure showing the segment of the angular artery affected by the atherosclerosis (black arrow).

After antisepsis of the surgical area, local anesthesia with 2% lidocaine with 1:100,000 epinephrine was applied. Next, the skin was incised with No. 15 scalpel blade and dissected with Metzenbaum scissors to the muscular plane. During the intraoperative exploration, the surgeon noted that a segment of the angular artery at its junction with the superior labial one was hardened, and no epidermoid cyst was detected (Figure 2). The stiffened segment of the angular artery was excised, and its end-to-end extremities were ligated with an absorbable suture. Following hemostasis, the skin was sutured in layers. The specimens were immediately immersed in buffered 10% formaldehyde solution and sent to a pathology laboratory for histopathological examination. The patient was discharged and returned 1 week later with no symptoms and a well-healed wound skin.

On the macroscopy view, a soft tissue fragment presenting an irregular shape, brownish color, and rubbery consistency measuring 1.2 × 1.2 × 0.7 cm was noticed. From this main fragment, there was a small, elongated, cylindrical, and stiff segment with a blood vessel-like appearance. Thus, the specimen was transversally cut, and the fragments were routinely processed and embedded in paraffin. The 5-*μ*m-thick tissue section was obtained and stained with hematoxylin–eosin (H&E).

Microscopically, H&E-stained sections of a muscular artery clearly showed an intima layer thickening associated with fibrosis, mild inflammation, and patches of cholesterol-containing plaques, but cholesterol crystals were not evidenced. Some atheromatous plaques also had an accumulation of foam cells (lipid-containing macrophages) with bubble-like cytoplasmatic appearance and smooth muscle cells. Moreover, several deposits of basophilic calcifications of different sizes were demonstrated mainly inside the intima, with some deposits also compromising the tunica media. In some fragments, such deposits either extended around the vessel's circumference or were limited to a small portion of such layers. Others were seen near the internal elastic fibers, focally causing its disruption. Necrosis was not detected. The tunica media and adventitia were entirely preserved.

**Figure 3:**
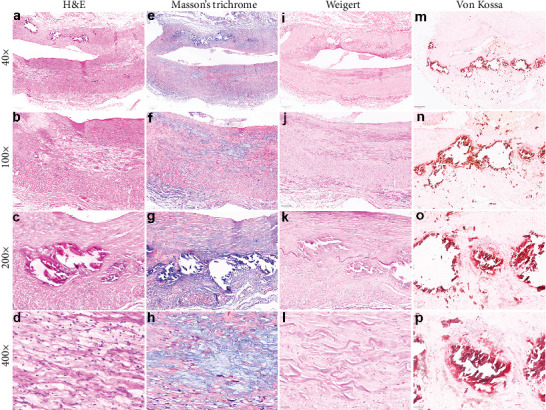
(a–d) The H&E staining reveals a notable thickening of the intima, accompanied by the presence of cholesterol accumulations containing foam cells and a minor population of smooth muscle cells. Additionally, deposits of dystrophic calcification are evident. (e–h) Masson's trichrome stain reveals extensive accumulations of collagen fibers within the intima layer and in the vicinity of dystrophic calcifications. (i–l) Weigert stain illustrates the presence of elastic fibers within the intima layer and in the region where the internal elastic lamina appears to be disrupted by dystrophic calcification. (m–p) Von Kossa stain highlights calcium deposits within the intima layer.

Based on the H&E findings, special stains, including Masson's trichome for connective tissue, Weigert for elastic fibers, and Von Kossa silver nitrate for calcium deposits, were employed to characterize the lesion morphologically. Masson's trichrome stain showed extensive areas of fibrosis in the intima layer (Figure 3(e–h)). Weigert stain showed several elastic fibers in the intima and the inner elastic fiber almost entirely preserved, except in some regions where calcified deposits provoked this disruption (Figure 3(i–l)). Finally, the Von Kossa stain confirmed calcium-associated deposits in the tunica intima and a few in the tunica media (Figure 3(m–p)). The final diagnosis of atherosclerosis of the angular artery, a branch of the facial artery, was made.

**Figure 4:**
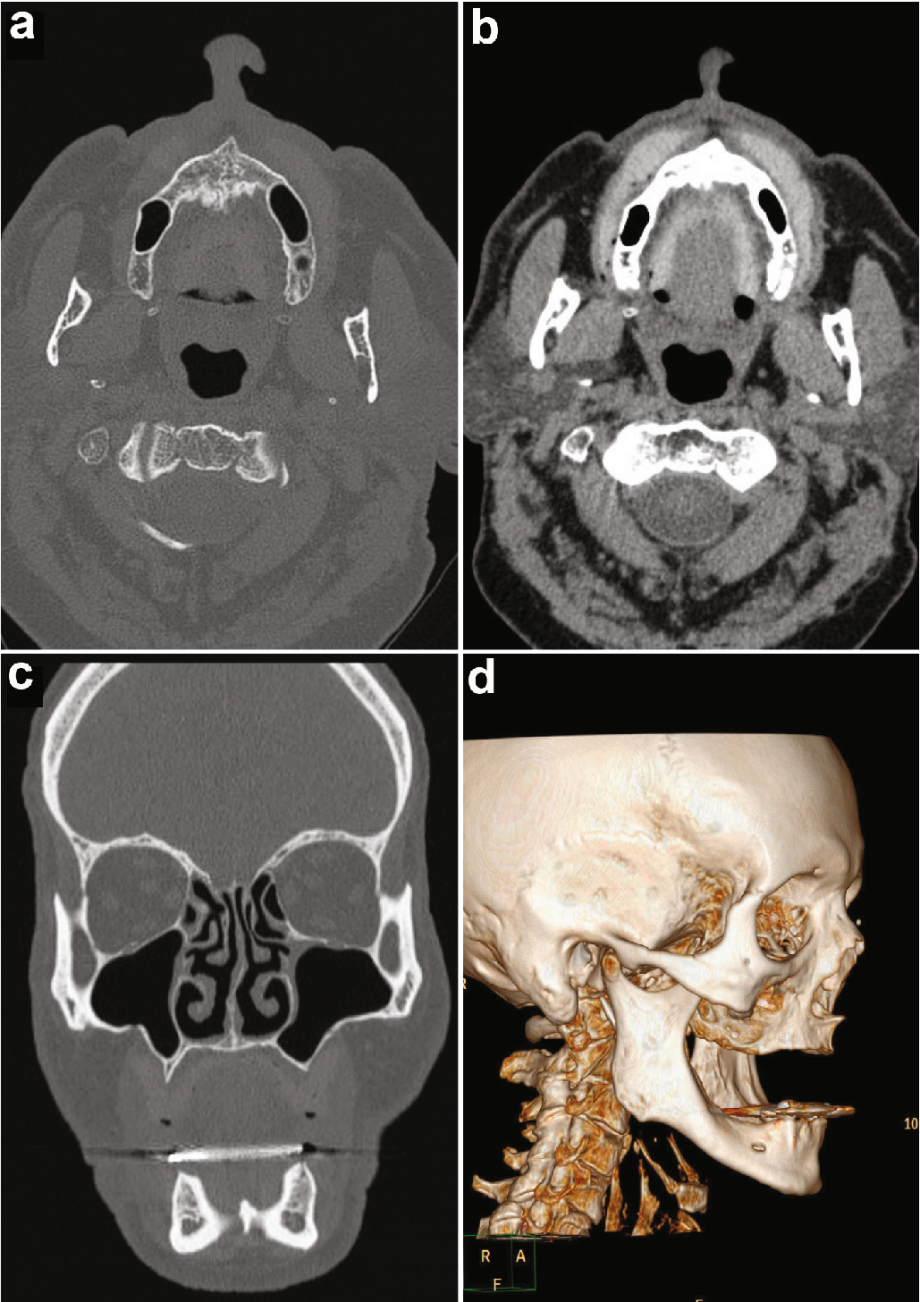
Multislice computed tomography examination of the patient showing no signs of the disease after lesion removal and the absence of other atherosclerotic changes in the head and neck. (a) Axial section of the upper jaw. (b) Soft tissue window of the axial section of the upper jaw. (c) Coronal section. (d) Three-dimensional reconstruction.

After establishing such a diagnosis, the surgeon asked the patient to undergo a computerized tomography (CT) scan of the head and neck to possibly detect alterations in other facial artery branches. The CT scan did not detect alterations in other facial artery branches (Figure 4). Since the diagnosis, the dentist and cardiologist have routinely monitored him. No recurrence or involvement of other facial artery branches was noticed after 6 months of follow-up.

### 2.1. Literature Review

An English literature review was performed using the PubMed and Scopus databases to identify only case reports of the atherosclerosis-affected facial arteries and their branches with no start date restriction until 2024. The following search terms were used: “atheroma” OR “atherosclerosis” AND “facial artery” OR “angular artery.” From the retrieved articles, we also performed a cross-referencing to find others. All articles showing clinicopathologic features with a histological-based diagnosis of atherosclerosis of the facial arteries and their branches were included. On the other hand, studies reporting inconsistent or equivocal features, absence or suboptimal histologic illustration, or vague clinicopathologic data were excluded. Sociodemographic characteristics, clinical features, location, and treatment were collected and tabulated for each case report.

Only five case reports were retrieved from the English literature.  Table 1 depicts the main aspects of the lesions collected from the selected articles, including ours. Briefly, four patients were males, and two were females. The median age of the patients was 61.3 years, ranging from 43 to 86 years old, supporting the prevalence of the lesion in older patients. The facial artery was the most common artery involved, followed by the labial one. All patients, but one, had a history of chronic systemic disease, being hypertension the most frequent underlying disease. In all cases, the lesion was removed surgically. Interestingly, three case reports had a diagnosis of atherosclerosis-associated aneurysm of the compromised artery.

## 3. Discussion

Atherosclerotic cardiovascular is one of the major causes of death worldwide, with at least 20% of them caused by thromboembolism-associated ischemic stroke from the external and internal carotid artery–derived atheromatous plaques [15, 19]. Here, we described a case of atherosclerosis affecting an extracranial head and neck artery in an elderly patient. It represents a unique case since no previous case report of atherosclerotic plaque–affected angular artery, a branch of the facial artery, was published in the literature.

Atherosclerosis of the external carotid–branched arteries is extremely rare, with only five reported cases published in the literature [13, 15–18]. The reason for the rarity of this particular artery being affected by the disease is currently unknown. However, it may be hypothesized that either the differential hemodynamics observed in the arterial tree or the distinction of regional development among arteries throughout the body could play a role [20]. In this location, the commonest artery compromised by such a disease is the facial artery (three cases), followed by the labial one (two cases). The major risk factors for developing such lesions seem to be the same as observed in other large- and medium-sized arteries of the body, including diabetes, hypertension, and dyslipidemia [9, 13]. Most of the affected patients, including ours, suffered from chronic diseases, especially hypertension. In only one case, the lesion developed in a middle-aged woman with no medical history of chronic cardiovascular disease [15]. Concerning our patient, his medical history of hypertension may have contributed to atherosclerosis development in the angular artery.

Clinically, these lesions manifest as slow-growing, painless swelling, or tumor-like masses [15, 17]. In our case, the patient reported a painless, firm, nodular lesion of 5 years duration in the skin closed to the labial commissure, leading to the initial diagnosis of an epidermoid cyst. Complementary to the clinical diagnosis, CT scan, magnetic resonance imaging, and angiography may be employed to confirm the presence of atheroma [11, 15]. However, as there was not any clinical suspicion of vascular or tumor-like disease in our case, an imaginological examination was not employed before the surgery. Indeed, it was only during the surgical procedure that the surgeon noticed the vascular origin of the lesion, which was confirmed histopathologically. Then, shortly after the final diagnosis of atherosclerosis, a CT scan was performed, but it did not detect any alteration in other facial-branched arteries.

Histopathologically, atherosclerotic lesions are comprised of cholesterol- and cholesterol ester–laden lipid core, fibrosis, and inflammation of the muscular- and elastic-derived intima layer [7]. Moreover, some cells, including smooth muscle, macrophages (known as foam cells), and T lymphocytes, are also components of such lesions [21]. Necrotic debris and necrosis-associated dystrophic calcification can also be seen [22]. Concerning our case, all such features, but necrosis, were easily observed microscopically. Also, the special stains employed, especially Masson's trichome and Weigert, highlighted a large deposition of collagen and elastic fibers in the intima, respectively. Von Kossa silver nitrate stain also confirmed calcium deposits in the artery's wall. As far as we are concerned, this is the first report in the literature that describes the morphological aspects of atherosclerotic plaques in a facial artery–branched angular artery.

Interestingly, in the literature review, it was found three case reports in which the diagnosis of atherosclerosis-associated aneurysm of the compromised facial and labial artery walls was made, a feature that was not observed in our case [13, 16, 17]. Regarding the external carotid–branched arteries, true aneurysms are extremely rare and most of them are atherosclerosis-associated artery wall dilation of the affected blood vessel, while others may have a congenital etiology [13, 23–25]. Indeed, it is well known that aneurysm may be one of the major consequences of elastic- and muscular artery–derived atherosclerotic lesions with increased risk of rupture and hemorrhage [24]. The affected patients of such works did not have any episodes of local hemorrhage. However, it is still unclear why the facial artery is one of the most affected extracranial head and neck blood vessels with atherosclerosis-associated aneurysms.

The treatment option is surgical excision of the artery's segment compromised by the lesion, followed by the ligation of its extremities with suture. All case reports found in the literature were treated surgically, including ours. Despite the fact that the consequences of atherosclerosis, including stroke, thromboembolism, and ischemia, are more associated when it develops in the internal carotids, its occurrence in the facial artery and its branches does not seem to be linked to such complications other than swelling or, at least, has not been hitherto reported in the literature [15]. Likewise, recurrence is not expected to happen, although we cannot exclude the probability of other facial-branched arteries being further affected by the lesion [15]. All cases retrieved from the literature, including ours, had no history of recurrence or the development of new lesions in another artery.

This report adds the sixth case of atherosclerosis of the extracranial head and neck arteries and the first one affecting the angular artery, a facial artery branch. Despite its rarity, clinicians should include atherosclerosis in the differential diagnosis of nodular or tumor-like masses in the face of chronic cardiovascular disease–harboring elderly patients.

## Figures and Tables

**Table 1:** Summary of previous case reports of atherosclerosis of the facial arteries and their branches.

**Case**	**Author (year)**	**Age (years)**	**Gender**	**Location**	**Size (mm)**	**Clinical aspects**	**Artery affected**	**Systemic disease**	**Treatment**	**Final diagnosis**
1	Quinn (1978) [17]	60	Female	Lower lip mucosa	4	Lump	Labial	NI^a^	Surgical removal	Aneurismal atherosclerosis
2	Galindo et al. (2006) [16]	60	Male	Right facial region	1.5	Mass	Facial	Hypertension, diabetes, and hypercholesterolemia	Surgical removal	Aneurismal atherosclerosis
3	Hoshi et al. (2010) [13]	73	Male	Right mandibular triangle	2.0	Swelling	Facial	No	Surgical removal	Aneurismal atherosclerosis
4	J. M. Kim, C. D. Kim, and S. W. Kim (2012) [18]	46	Male	Upper lip and right lateral side lip	1.0 and 1.5	Masses	Labial	Hypertension	Surgical removal	Atherosclerosis
5	Lee et al. (2016) [15]	43	Female	Right infra-auricular	2.0	Mass	Facial	No	Surgical removal	Atherosclerosis
6	Our case	86	Male	Right labial commissure	1.2	Nodule	Angular	Hypertension	Surgical removal	Atherosclerosis

^a^Not informed.

## Data Availability

The data that support the findings of this study are available from the corresponding author upon reasonable request.
